# 

**Published:** 1903-09

**Authors:** 


### Austin District Medical Society.

---

The fifty-fifth quarterly meeting of the Austin District Medical Society will be held in Austin, Texas, Tuesday, September 29, 1903.

The following papers will be presented:

1. "A Case of Hepatic Abscess," by Dr. Sam Haigler, Austin.
2. "The Value of Modern Aids to Diagnosis of Diseases of the Stomach," by Dr. J. W. McLaughlin, Galveston.
3. "Use of Streptolytic Serum in Mixed Infection of Tuberculosis," by Dr. H. N. Graves, Georgetown.
4. "Some Remarks on Operation for Inguinal Hernia," by Dr. T. J. Bennett, Austin.
5. "Intra-Spinal Injection of Tetanic Serum in Tetanus," by Dr. A. Garwood, New Braunfels.
6. "The Influence of Higher Education on Womanhood and Motherhood," by Dr. J. W. Carhart.

Voluntary papers.

A general clinic will be held. It is expected that the clinic will prove to be a very important feature of the program, as a number of the members of the profession have promised to have patients present.

Be with us on the 29th.

F. E. DANIEL, President.

W. A. HARPER, Secretary.

